# The development of polycythemia vera after phlebotomy treatment for hereditary haemochromatosis

**DOI:** 10.1002/jha2.222

**Published:** 2021-05-21

**Authors:** Erik JM van Bommel, Denise Kelder, Marlijn PA Hoeks, Alexandra HE Herbers

**Affiliations:** ^1^ Department of Internal Medicine/Haematology Jeroen Bosch Hospital ‘s‐Hertogenbosch The Netherlands; ^2^ Department of haematology Radboud University Medical Center Nijmegen The Netherlands; ^3^ Unit Transfusion Medicine Sanquin blood bank Amsterdam The Netherlands

## 1 INTRODUCTION

Hereditary haemochromatosis (HH) is an autosomal recessive disorder, related to mutations in the *HFE* gene, characterized by increased intestinal iron absorption through decreased hepcidin levels. It is one of the most common genetic disorders in individuals of European descent and can lead to clinically manifest iron overload. In turn, HH can lead to several complications, including diabetes and liver or heart failure [1]. The majority of patients with the myeloproliferative neoplasm (MPN) polycythemia vera (PV), which is characterized by erythrocytosis, harbour a janus kinase 2 (JAK2) mutation [2]. The co‐existence of HH and PV is rare. As such, relatively small genetic studies did not reveal HH mutations in a PV population (52 patients) [3], nor were there any significant associations between the occurrence of different haematological malignancies (including 15 PV patients) and *HFE* mutations [4].

## 2 CASE

Here, we present a case where the two conditions co‐exist. An otherwise healthy 68‐year‐old male with HH (homozygous *HFE C282Y* mutation) and secondary liver cirrhosis was treated with regular phlebotomies in order to prevent further liver dysfunction. He smoked five cigarettes/day until 1990. When the phlebotomies commenced in 2018, his haemoglobin level was 174 g/L with a haematocrit of 46%, and his serum ferritin level was 5000 μg/L with a transferrin saturation of 84%. Phlebotomies were halted in January 2020 when his serum ferritin level fell to 11 μg/L. At follow‐up in July 2020, his serum ferritin level and transferrin saturation had further dropped to 7.8 μg/L and 3%, respectively. This could not be explained by phlebotomies or blood loss otherwise. Surprisingly, in this same period, his haemoglobin level and haematocrit rose from 151 g/L to 185 g/L and from 42% to 64%, respectively. Additional testing revealed that serum erythropoietin was low (<1.0 IU/L), while bone marrow analysis showed increased erythropoiesis and megakaryopoiesis. Although *JAK2 V617F* analysis was inconclusive, a *JAK2 exon 12* mutation was present, rendering all (major and minor) diagnostic WHO criteria for PV [5]. Phlebotomies were resumed and hydroxycarbamide was initiated to achieve cytoreduction. Furthermore, acetylsalicylic acid and allopurinol (uric acid 0.63 mmol/L) were initiated in order to prevent ischaemic events and hyperuricemia/gout.

## 3 DISCUSSION

Our case is fairly similar to one that was previously described [6]. There, the authors viewed the co‐occurrence of HH and PV as an unrelated and rare coincidence, based on the above mentioned genetic studies [6]. However, Andrikovics et al found decreased allele frequencies of genetic *HFE* and *TFR* variants with a casual role in iron overload in MPN patients, including 175 PV patients, compared with healthy blood donors [7], indicating a protective role for iron overload against MPN. In addition, iron deficiency and anaemia have been suggested to evoke PV. This is supported by the observation that former blood donors were about three times more prevalent in a studied PV cohort than in the general population [8].

We retrospectively performed genetic testing on a blood sample that was obtained from our patient before phlebotomy treatments commenced in 2018 and found that the JAK2 exon 12 mutation was already present. We therefore hypothesize that HH‐related iron overload is toxic for the JAK2 clone and hinders clinical manifestation of the PV phenotype. When we removed this impediment by starting phlebotomies, it allowed the PV phenotype to surface. We believe our case provides a strong clue for the fact that HH and PV are not unrelated. In fact, HH‐related iron overload seems to be an important disease modifier of PV.

## AUTHOR CONTRIBUTIONS

EJM van Bommel wrote the first draft of this manuscript. D Kelder, MPA Hoeks and AHE Herbers reviewed and corrected this draft and approved the submitted version.

## CONFLICT OF INTEREST

The authors declare that there is no conflict of interest that could be perceived as prejudicing the impartiality of the research reported.

## References

[1] Feder JN , Gnirke A , Thomas W , Tsuchihashi Z , Ruddy DA , Basava A , et al. A novel MHC class I‐like gene is mutated in patients with hereditary haemochromatosis. Nat Genet. 1996;13(4):399–408. 8696333 10.1038/ng0896-399

[2] Wang YL , Vandris K , Jones A , Cross NC , Christos P , Adriano F , et al. JAK2 Mutations are present in all cases of polycythemia vera. Leukemia. 2008;22(6):1289. 18079740 10.1038/sj.leu.2405047

[3] Franchini M , de Matteis G , Federici F , Solero P , Veneri D . Analysis of hemochromatosis gene mutations in 52 consecutive patients with polycythemia vera. Hematology. 2004;9(5‐6):413–4. 15763983 10.1080/10245330400001934

[4] Hannuksela J , Savolainen ER , Koistinen P , Parkkila S . Prevalence of HFE genotypes, C282Y and H63D, in patients with hematologic disorders. Haematologica. 2002;87(2):131–5. 11836162

[5] Arber DA , Orazi A , Hasserjian R , Thiele J , Borowitz MJ , Le Beau MM , et al. The 2016 revision to the World Health Organization classification of myeloid neoplasms and acute leukemia. Blood. 2016;127(20):2391–405. 27069254 10.1182/blood-2016-03-643544

[6] Singh P , Toom S , Shrivastava MS , Solomon WB . A rare combination of genetic mutations in an elderly female: a diagnostic dilemma! Blood. 2016;128(22):5487.

[7] Andrikovics H , Meggyesi N , Szilvasi A , Tamaska J , Halm G , Lueff S , et al. HFE C282Y mutation as a genetic modifier influencing disease susceptibility for chronic myeloproliferative disease. Cancer Epidemiol Biomarkers Prev. 2009;18(3):929–34. 19258483 10.1158/1055-9965.EPI-08-0359

[8] Najean Y , Rain JD , Billotey C . Epidemiological data in polycythaemia vera: a study of 842 cases. Hematol Cell Ther. 1998;40(4):159–65. 9766920

